# Bone Fracture Detection Using Deep Supervised Learning from Radiological Images: A Paradigm Shift

**DOI:** 10.3390/diagnostics12102420

**Published:** 2022-10-07

**Authors:** Tanushree Meena, Sudipta Roy

**Affiliations:** Artificial Intelligence & Data Science, Jio Institute, Navi Mumbai 410206, India

**Keywords:** artificial intelligence, bone imaging, computer vision, deep learning, fractures, radiology

## Abstract

Bone diseases are common and can result in various musculoskeletal conditions (MC). An estimated 1.71 billion patients suffer from musculoskeletal problems worldwide. Apart from musculoskeletal fractures, femoral neck injuries, knee osteoarthritis, and fractures are very common bone diseases, and the rate is expected to double in the next 30 years. Therefore, proper and timely diagnosis and treatment of a fractured patient are crucial. Contrastingly, missed fractures are a common prognosis failure in accidents and emergencies. This causes complications and delays in patients’ treatment and care. These days, artificial intelligence (AI) and, more specifically, deep learning (DL) are receiving significant attention to assist radiologists in bone fracture detection. DL can be widely used in medical image analysis. Some studies in traumatology and orthopaedics have shown the use and potential of DL in diagnosing fractures and diseases from radiographs. In this systematic review, we provide an overview of the use of DL in bone imaging to help radiologists to detect various abnormalities, particularly fractures. We have also discussed the challenges and problems faced in the DL-based method, and the future of DL in bone imaging.

## 1. Introduction

**Figure 1:**
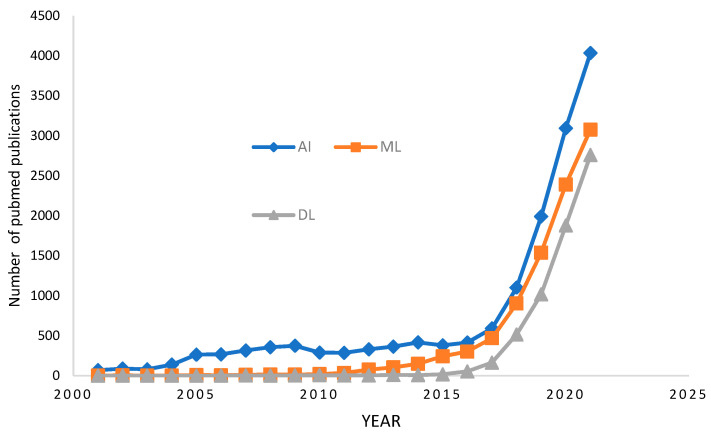
The number of papers on PubMed when searching on the phrases “radiology” with “artificial intelligence,” “machine learning,” or “deep learning” reflects the growth of AI in radiography.

In radiology, AI is being used for various tasks, including automated disease detection, classification, segmentation, quantification, and many other works. Research shows that deep learning (DL), a specific subset of artificial intelligence (AI), can detect diseases more accurately than medical practitioners from medical images [1]. Bone Magnetic Resonance Imaging (Bone MRI), X-ray, and Computerized Tomography (CT) are the common key area among DL in medical imaging research. We need advanced and compatible DL methods to exploit bone imaging-specific data since the tremendous volume of data is burdensome for physicians or medical practitioners. The increasing amount of literature on this domain reflects the high level of interest in developing AI systems in radiology. About ten years earlier, the maximum count of AI-related publications in radiology each year hardly exceeded 100. Later, we witnessed enormous growth, with annual publications ranging from 1000 to 2000. The number of papers on AI in radiology on PubMed is reflected in Figure 1. DL is used at the time of image capturing and restructuring to enhance the speed of acquisition, quality of image, and reduced cost. It can also denoise images, register them, and translate them between multiple modalities. Furthermore, many DL systems for medical image processing are developing, such as computer-aided diagnosis, segmentation, and anomaly detection.

Furthermore, different activities like annotation and data labelling are very much essential. However, inter-user, intra-user labelling, and different resolution and protocol variations are significant [2,3] due to varying experiences and varied settings. This can lead to noisy labelling. Therefore, standardising image labelling is still a great work in DL.

**Figure 2:**
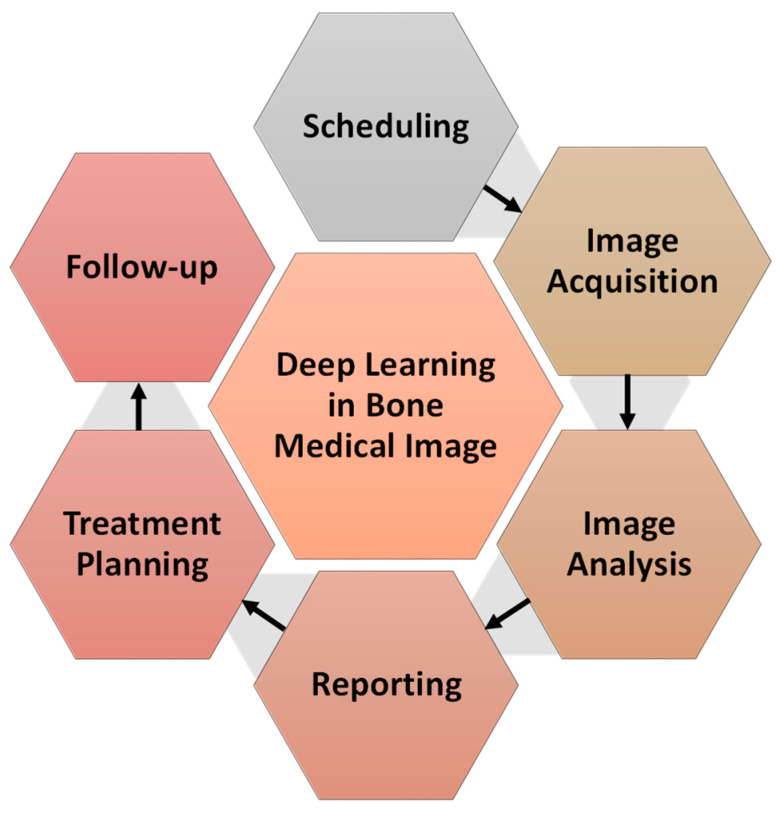
Illustrations of machine learning in radiology.

Currently, many AI techniques, in particular deep learning (DL) research, are going on to help medical practitioners by automating the image processing and analytics even in co-clinical trials, a process known as “computational radiology” [4]. Detection of clinical findings, identifying the illness extent, characterization of clinical conclusions (e.g., into malignant and benign tissue), and various software techniques, which can be widely referred to as decision support systems, are all examples of computerised tools that can be built. Due to time constraints and a lack of visualisation and quantification capabilities, these are rarely incorporated in today’s radiological reports. The various illustrations of DL in bone radiology are shown in Figure 2.

This article gives an overall view of the use of DL in bone fracture detection. Additionally, we have presented the future use of DL in radiology.

An extensive systematic review search was conducted regarding deep learning in fracture detection. This is a retrospective study that combines and interprets the acquired data. All articles were retrieved from PubMed, Elsevier, and radiology library databases. The keywords used to search the articles are the combination of the words like “deep learning or machine learning in fracture detection” or “artificial intelligence in fracture diagnosis” or “neural network in fracture detection”. The search was conducted in March 2022.

The following key questions are answered in this review:

- What different kinds of bone fractures are there?
- What deep learning techniques are used in bone fracture detection and classification?
- How are deep learning methods beneficial over traditional methods?
- How does deep learning in radiology immensely help medical practitioners?
- What are the current challenges and opportunities in computerized disease detection from the bone?
- What are the future research prospects in this field?

### 1.1. Common Bone Disorder

Orthopaedicians and radiologists use X-ray images, MRI, or CT of the injured bone to detect a bone abnormality. In recent years, there has been a significant advancement in the development of DL, making it possible to deploy and evaluate deep learning models in the medical industry. Musculoskeletal is one of the biggest problems in orthopaedics. Musculoskeletal diseases include arthritis, bursitis, tendinitis, and several others. In the short term, they cause severe pain, whereas, in the long term, the pain gets more severe and sometimes can even lead to disabilities. Therefore, early detection is very important using DL.

**Figure 3:**
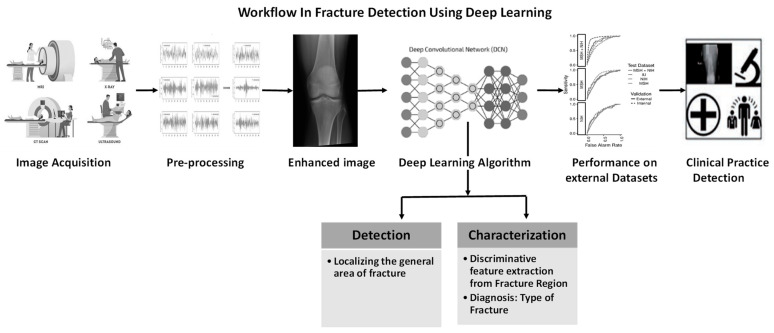
Workflow in fracture detection using deep learning.

Bone fracture from X-ray is another one of the most common injuries these days. Every year, the number of fractures occurring across the EU6 nations, Sweden, Germany, Italy, France, Spain, and the UK, is 2.7 million [5]. Doctors face various difficulties in evaluating X-ray images for several reasons: first, X-rays may obscure certain bone traits; second, a great deal of expertise is required to appropriately diagnose different forms of fractures; and third, doctors frequently have emergency situations and may be fatigued. It was observed that the efficiency of radiologists in the evaluation of musculoskeletal radiographs reduces by the end of the workday compared to the beginning of the workday in detecting fractures [6]. In addition, radiographic interpretation often takes place in environments without the availability of qualified colleagues for second opinions [7]. The success of the treatment and prognosis strongly depends on the accurate classification of the fracture among standard types, such as those defined by the Arbeitsgemeinschaft für Osteosynthesefragen (AO) foundation. In that context, a CAD system that can help doctors might have a direct impact on the outcome of the patients. The possible solution for fracture detection using deep learning is shown in Figure 3.

### 1.2. Importance of Deep Learning in Orthopaedic and Radiology

The implementation of deep learning techniques in addition to traditional techniques in radiology offers the possibility to increase the speed and efficiency of diagnostic procedures and reduce the workload through transferring time-intensive procedures from radiologists to the system. However, deep learning algorithms are vulnerable to some of the drawbacks of inter- and intra-observer inconsistency. In the case of academic research, DL is performing exceptionally well, and it even surpasses human performance in detecting and classifying fractures from the radiographic images. In the past few years, deep learning has garnered a significant amount of interest among the researchers. Modern research has demonstrated that deep learning can execute difficult analyses at the level of medical experts [8]. Several research in orthopaedic traumatology have used deep learning in radiographs to diagnose and classify fractures [9,10]. However, deep learning in fracture detection on CT images, on the other hand, has received very little attention [11]. DL has very good potential to estimate the parameters for fracture detection like normal distal radius angles, radial inclination, radial height, palmar tilts, etc.

### 1.3. Historical Perspective

The breakthrough of DL methods came after CNN’s dominance of the ImageNet data set, which was demonstrated in a large-scale picture categorization challenge in 2012 [12]. At that time, DL was the most popular machine-learning technology, prompting a discussion in the medical imaging community about whether DL could be used in medical imaging. The discussion stemmed from the aforementioned issues known as the data challenge, with the biggest one being a lack of sufficient labelled data.

Numerous methods can be identified as enablers of DL methodology in the medical imaging area; methods were introduced in 2015–2016 that used “transfer learning” (TL) (also known as “learning from nonmedical features”) to utilise acquired experience from attempting to solve a reference problem to a separate but related target problem and used in bone imaging. This was demonstrated by several groups [13,14,15]; employing a fine-tuned deep network trained on ImageNet to a medical imaging problem statement accelerated training convergence and improved accuracy. Synthetic data augmentation evolved as an alternative approach for processing limited data sets in 2017–2018. Now, classical augmentation is considered as a crucial component of every network training. However, the significant challenges to be addressed were whether it would be feasible to synthesise medical data utilising techniques like generative modelling and whether the synthesised data would serve as legitimate medical models and, in reality, improve the execution of the medically assigned task. Synthetic image augmentation using the (GAN) was used in work [16] to produce lesion image samples that have not been identified as synthetic by skilled radiologists while simultaneously improving CNN performance in diagnosing liver lesions. GANs, vibrational encoders, and variations on these have been examined and progressed in recent research. The U-Net architecture [17] is one of the most important contributions from the community of medical imaging in terms of image segmentation of bone disease from images.

## 2. Deep Learning and Key Techniques in Bone Imaging

Clinical implications of high-quality image reconstruction from low dosages and/or quick acquisitions are significant. Clinical image improvement, which seeks to alter a pixel intensities such that the produced image is better suited for presentation or further study. MR bias field correction, denoising, super-resolution, and image harmonisation are some of the enhancement techniques. Modality translation and synthesis, which may be thought of as image-enhancing procedures, have received a lot of attention recently. Deep learning helps target lesion segmentations and clinical measurement, therapy, and surgical planning. DL-based registration is widely used in multimodal fusion, population, and longitudinal analysis, utilizing image segmentation via label transition. Other DL and pre-processing technologies include target and landmark detection, view or picture identification, and automatic report generation [18,19,20].

Deep learning models have a higher model capacity and generalization capabilities than simplistic neural network models. For a single task, deep learning methods trained on large datasets generate exceptional results, considerably surpassing standard algorithms and even abilities.

### 2.1. Network Structure

**Figure 4:**
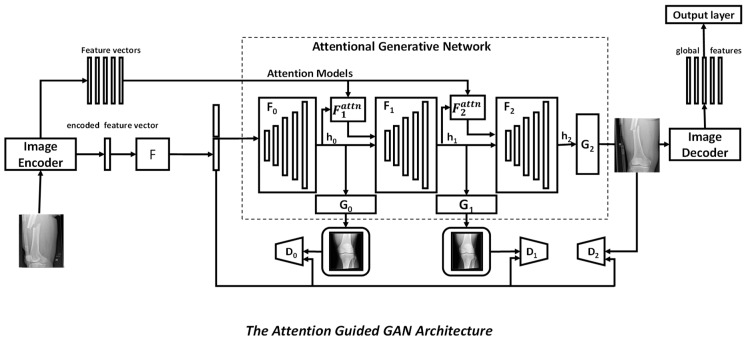
General architecture of attention guided GAN.

ResNet [21], VGGNet [22], and Inception Net [23] illustrate a research trend that started with AlexNet [12] to make networks deeper and used in abnormality visualization in bone. DenseNet [24,25] and U-Net [16] show that using skip connections facilitates a deep network that is even more advanced. The U-net was created to keep-up with segmentation, and other networks were developed to perform image classification. Deep supervision [26] boosts discriminative ability even more. Bone imaging, including medical image restoration, image quality improvement, and segmentation, makes extensive use of adversarial learning. When summarising bone image data or developing a systematic decision, the attention mechanism enables the automated detection of “where” and “what” to concentrate on. Squeeze and excitation are two mechanisms that can be used to control channel attention. In [27], attention is combined with the generative adversarial network (GAN), while in [28], attention is combined with U-Net. Figure 4 shows a general architecture of attention-guided GAN. GANs are adversarial networks consisting of two parts: a generator and a discriminator. A generator learns the important features and generates feature maps by mapping them to a latent space. The discriminator learns to distinguish the feature maps generated by the generator from the learnt true data distribution. Attention gates are involved in the pipeline in order to represent the important feature vectors efficiently without increasing the computation complexity. The learnt features, which are in the form of a distribution of raw vectors, are then fed into an image encoder. It is then fed into an output layer to provide the required output.

### 2.2. Annotation Efficient Approaches

**Figure 5:**
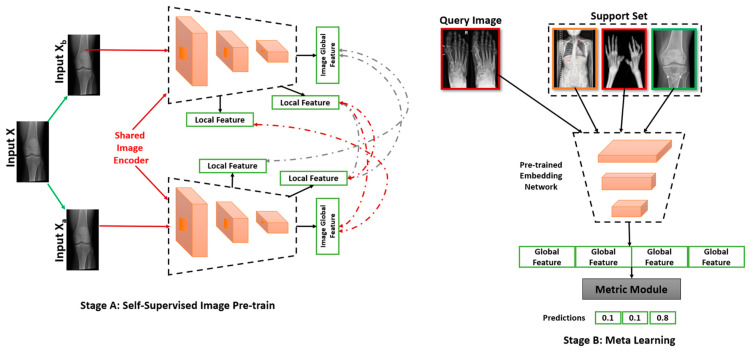
Diagrammatic representation of self-supervised learning.

DL in bone imaging need to adapt feature representation capacity obtained from existing models and data to the task at hand, even if the models and data are not strictly within the same domain or for the same purpose. This integrative learning process opens the prospect of working with various domains including multiple heterogeneous tasks for the first time. One possible approach to solve new feature learning is the self-supervised learning shown in Figure 5. In Figure 5A, the target images are shared with the image encoder wherein the encoder learns the important patterns/features by resampling them into a higher dimensional space. These learnt local features are then propagated to the output layer. The output layer may consist of a dense layer for classification or a 1 × 1 convolutional layer for segmentation/localisation of the object. Figure 5B is used for the transfer of the learnt features to a modified architecture to serve the purpose of reproducibility of the model. Multiple such learnt features are then collected as model weights which undergo an embedding network. This enables the model to perform multiple tasks at once via a multi-headed output.

### 2.3. Fractures in Upper Limbs

The probability of missing fractures between both the upper limbs and lower limbs is similar. The percentage of missing the fractures in the upper limbs consisting of the elbow, hand, wrist, and shoulder is 6%, 5.4%, 4.2%, and 1.9%, respectively [29]. Kim et al. [30] used 1112 wrist radiograph images to train the model, and then they incorporated 100 additional images (including 50 fractured and 50 normal images). They achieved diagnostic selectivity, sensitivity, and area under the curve (AUC) is 88%, 90% and 0.954, respectively. Lindsey et al. [8] used 135,409 radiographs to construct a CNN-based model for detecting wrist fractures. They claimed that the clinicians’ image reading (unaided and aided) improved from 88% to 94%, respectively, resulting in 53% reduction in misinterpretation. Olczak et al. [31] developed a model for distal radius fractures and evaluation was done on hand and wrist radiographic images. They analysed the network’s effectiveness with that of highly experienced orthopaedic surgeons and found that it has a good performance with sensitivity, and specificity of 90% and 88%, respectively. They did not define the nature of fracture or the complexity in detecting fractures.

Chung et al. [32] developed a CNN-based model for detecting the proximal humerus fracture and classifying the fractures according to the Neer’s classification with 1891 shoulder radiographic images. In comparison with specialists, the model had a high throughput precision and average area under the curve, which is 96% and 1, respectively. The sensitivity is 99% followed by specificity as 97%. However, the major challenge is the classification of fractures. The predicted accuracy ranged from 65–85%. Rayan et al. [9] introduced a framework with a multi-view strategy that replicates the radiologist when assessing several images of severe paediatric elbow fractures. The authors analysed 21,456 radiographic investigations comprising 58,817 elbow radiographic images. The accuracy sensitivity and specificity of the model are 88%, 91%, and 84%, respectively.

### 2.4. Fractures in Lower Limb

Hip fractures account for 20% of patients who are admitted for orthopaedic surgery, whereas occult fracture on radiographic images occurs at a rate ranging between 4% to 9% [10]. Urakawa et al. [10] designed a CNN-based model to investigate inter-trochanteric hip fractures. They used 3346 hip radiographic images, consisting of 1773 fractured and 1573 non-fractured radiographic hip images. The performance of the model was compared with the analysis of five orthopaedic surgeons. The model showed better performance than the orthopaedic surgeons. They reported an accuracy of 96% vs. 92%, specificities of 97% vs. 57% and sensitivities of 94% vs. 88%. Cheng et al. [33] used 25,505 pre-trained limb radiographic images to develop a CNN-based model. The accuracy of the model for detecting hip fractures is 91%, and its sensitivity is 98%. The model has a low false-negative rate of 2%, which makes it a better screening tool. In order to detect the femur fracture, Adams et al. [34] developed a model with a 91% accuracy and an AUC of 0.98. Balaji et al. [35] designed a CNN-based model for the diagnosis of femoral diaphyseal fracture. They used 175 radiographic images to train the model for the classification of the type of femoral diaphyseal fracture namely spiral, transverse, and comminuted. The accuracy of the model is 90.7%, followed by 92.3% specificity and 86.6% sensitivity.

Missed lower extremity fractures are common, particularly in traumatic patients. Recent studies show that the percentage of missed diagnoses due to various reasons is 44%, and the percentage of misdiagnoses because of radiologists is 66%. Therefore, researchers are trying hard to train models so that they can help radiologists in fracture detection more accurately. Kitamura et al. [36] proposed a CNN model using a very small number of ankle radiographic images (298 normal and 298 fractured images). The model was trained to detect proximal forefoot, midfoot, hindfoot, distal tibia, or distal fibula fractures. The model accuracy in fracture detection is from 76% to 81%. Pranata et al. [37] developed two CNN-based models by using CT radiographic images for the calcaneal fracture classification. The proposed model shows an accuracy of 0.793, specificity of 0.729, and sensitivity of 0.829, which makes it a promising tool to be used in computerized diagnosis in future. Rahmaniar et al. [26] proposed a computer-aided approach for detecting calcaneal fractures in CT scans. They used the Sanders system for the classification of fracture, in which calcaneus fragments were recognised and labelled using colour segmentation. The accuracy of the model is 86%.

### 2.5. Vertebrae Fractures

Studies show that the frequency of undiagnosed spine fractures is ranging from 19.5% to 45%. Burns et al. [38] used lumbar and thoracic CT images and were able to identify, locate, and categorise vertebral spine fractures along with evaluating the bone density of lumbar vertebrae. For compression fracture identification and localisation, the sensitivity achieved was 0.957, with a false-positive rate of 0.29 per patient. Tomita et al. [39] proposed a CNN model to extract radiographic characteristics from CT scans of osteoporotic vertebral fractures. The network is trained with 1432 CT images, consisting of 10,546 sagittal views, and it attained 89.2% accuracy. Muehlematter et al. [38] developed a model using 58 CT images of patients with confirmed fractures caused because of vertebral insufficiency to identify vertebrae vulnerable to fracture. The study included a total of 120 items (60 healthy and 60 unhealthy vertebrae). Yet, the accuracy of healthy/unhealthy vertebrae was poor with an AUC of 0.5.

## 3. Deep Learning in Fracture Detection

Various research has shown that deep learning can be used to diagnose fractures. The authors [30] were focused on determining how transfer learning can be used to detect fractures automatically from plain wrist radiographs pre-trained on non-medical radiographs, from a deep Convolutional Neural Network (CNN). Inception version 3 CNN was initially developed for the ImageNet Large Visual Recognition Challenge and used non-radiographical images to train the model [40]. They re-trained the top layer of the inception V3 model to deal with the challenge of binary classification using a training data set of about 1389 radiographs, which are manually labelled. On the test dataset of about 139 radiographs, they attained an AUC of 0.95. This proved that a CNN model trained on non-medical images could effectively be used for the fracture detection problem on the plain radiographs. They achieved the sensitivity and Specificity around 0.88 and 0.90, respectively. The degree of accuracy outperforms the existing computational models for automatic fracture detection like edge recognition, segmentation, and feature extraction (the sensitivities and specificities reported in the studies range between 75%–85%). Though the work offers a proof of concept, it has many limitations. During the training procedure, a discrepancy was observed between the validation and the training accuracy. This is probably due to overfitting. Various strategies can be implemented to reduce overfitting. One approach would be to apply automated segmentation of the most relevant feature map. Pixels beyond the feature map would be clipped from the image so that irrelevant features did not impact the training process. Lindsey et al. [8] propose another way to reduce overfitting: the use of advanced augmentation techniques. Another strategy used by [8] to minimize overfitting would be the introduction of advanced augmentation techniques. Additionally, in the field of machine learning, the study size of the population is sometimes a limiting constraint. A bigger sample size provides a more precise representation of the true demographics.

Chung et al. [32] used plain anterio-posterior (AP) shoulder radiographs to test deep learning’s ability of detecting and categorising proximal humerus fractures. The results obtained from the deep CNN model were compared with the professional opinions (orthopaedic surgeons, general physicians, and radiologists). There were 1891 plain shoulders AP radiographic images in their dataset. They applied a ResNet-152 model that has been re-modelled to their proximal humerus fracture samples. The performance of the CNN model to identify normal shoulders and fractured one was very high. Furthermore, significant findings for identifying the type of fracture based on plain AP shoulder radiographs were observed. The CNN model performed better than the general orthopaedic surgeons, physicians and shoulder specialized orthopaedic surgeons. This refers to the possibility of computer-aided diagnosis and classification of fractures and other musculoskeletal disorders using plain radiographic images.

Tomita et al. [39] performed retrospective research to assess the potential of DL to diagnose osteoporotic vertebral fractures (OVF) from CT scans and proposed an ML-based system, entirely driven by deep neural network architecture, to detect OVFs from CT scans. They employed a system with two primary components for their OVF detection system: (1) a convolutional neural network-based feature extraction module and (2) an RNN module to combine the acquired characteristics and provide the final evaluation. They applied a deep residual model (ResNet) to analyse and extract attributes from CT images. Their training, validation and testing dataset consisted of 1168 CT scans, 135 CT scans and 129 CT scans, respectively. The efficiency of their proposed methodology on an independent test set is equivalent to the level of performance of practising radiologists in terms of accuracy and F1 score. This automated detection model has the ability to minimise the time and manual effort of OVF screenings on radiologists. It also reduces false-negative occurrences in asymptomatic early stage vertebral fracture diagnosis.

**Figure 6:**
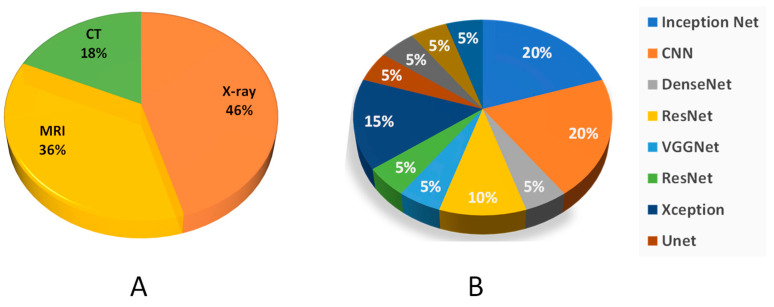
(**A**) Represents the usage of different modalities in deep learning. (**B**) Usage of deep learning models for fracture detection.

CT scan images were used as input images for the detection of cervical spine fractures. Since a CT scan provides more pixel information than X-rays, therefore the pre-processing techniques such as windowing and Hounsfield Unit conversion were used for easier detection of anomalies in the target tissues. However, the usage of X-ray images as input for the detection of lower and upper limb fractures results in the loss of vital pixel information, resulting in lower accuracies than the reported accuracies for cervical spine fractures. In terms of lower and upper limbs, upper limbs have greater bone densities, especially in the femur, enabling them to bear the entire body load. Figure 6 shows the pie plot of the usage of different modalities and different deep learning models. A summary of clinical studies involving computer-aided fracture detection and their reported success is given in Table 1 below.

## 4. Barriers to DL in Radiology & Challenges

There is widespread consensus that deep learning might play a part in the future practice of radiology, especially X-rays and MRI. Many believe that deep learning methods will perform the regular tasks, enabling radiologists to focus on complex intellectual problems. Others predict that radiologists and deep learning algorithms will collaborate to offer efficiency that is better than either separately. Lastly, some assume that deep learning algorithms will entirely replace radiologists. The implementation of deep learning in radiology will pose a lot of barriers. Some of them are described below.

### 4.1. Challenges in Data Acquisition

First, and foremost is the technical challenge. While deep learning has demonstrated remarkable promises in other image-related tasks, the achievements in radiology are still far from indicating that deep learning algorithms can supersede radiologists. The accessibility of huge medical data in the radiographic domain provides enormous potential for artificial intelligence-based training, however, this information requires a “curation” method wherein the data is organised by patient cohort studies, divided to obtain the area of concern for artificial intelligence-based analysis, filtered to measure the validity of capture and representations, and so forth.

However, annotating the dataset is time-consuming and labour-demanding, and the verification of ground truth diagnosis should be extremely robust. Rare findings are a point of weakness that is if a condition or finding is extremely rare, obtaining sufficient samples to train the algorithm for identifying it with confidence becomes a challenging task. In certain cases, the algorithm can consider noise as an abnormality, which can lead to inadvertent overfitting. Furthermore, if the training dataset has inherent biases (e.g., ethnic-, age- or gender-based), the algorithm may underfit findings from data derived from a different patient population.

### 4.2. Legal and Ethical Challenges

Another challenge is who will take the responsibility for the errors that a machine will make. This is very difficult to answer. When other technologies like elevators and automobiles were introduced, similar issues were raised. Considering artificial intelligence may influence several aspects of human activity, problems of this kind will be researched, and answers to these will be proposed in the future years. Humans would like to see Isaac Asimov’s hypothetical three principles of robotics implemented to AI in radiography, where the “robot” is an “AI medical imaging system.” Asimov’s Three Laws are as follows:

- A robot may not injure a human being or, through inaction, allow a human being to come to harm.
- A robot must obey the orders given it by human beings except where such orders would conflict with the First Law.
- A robot must protect its own existence as long as such protection does not conflict with the First or Second Laws.

The first law conveys that DL tools can make the best feasible identification of disease, which can enhance medical care; however, computer inefficiency or failure or inaction may lead to medical error, which can further risk a patient’s life. The second law conveys that in order to achieve suitable and clinically applicable outputs, DL must be trained properly, and a radiologist should monitor the process of learning of any artificial intelligence system. The third law could be an issue while considering any unavoidable and eventual failure of any DL systems. Scanning technology is evolving at such a rapid pace that training the DL system with particular image sequences may be inadequate if a new modality or advancement in the existing modalities like X-ray, MRI, CT, Nuclear Medicine, etc., are deployed into clinical use.

However, Asimov’s laws are fictitious, and no regulatory authority has absolute power or authority over whether or not they are incorporated in any particular DL system. Meantime, we trust in the ethical conduct of software engineers to ensure that DL systems behave and function according to adequate norms. When an DL system is deployed in clinical care, it must be regulated in a standard way, just like any other medical equipment or product, as specified by the EU Medical Device Regulation 2017 or FDA (in the United States). We can only ensure patient safety when DL is used to diagnose patients by applying the same high rules of effectiveness, accountability, and therapeutic usefulness that would be applied to a new medicine or technology.

### 4.3. Requirement for Accessing to Large Volumes of Medical Data

Access to a huge amount of medical data is required to design and train deep learning models. This could be a major limitation for the design of deep learning models. To collect training data sets, software developers use various methods; some collaborate directly with patients, while others engage with academic or institutional repositories. The information of every patient used by third parties must provide an agreement for use, and approval may be collected again if the data is again used in some other context. Furthermore, the copyright of radiology information differs by region. In several nations, the patient retains the ultimate ownership of his private information. However, it could be maintained in a hospital or radiology repository, provided they must have the patient’s consent. Data anonymization should be ensured. This incorporates much more than de-identification and therefore should confirm that the patient cannot be re-identified using DICOM labels, surveillance systems, etc.

## 5. Limitations and Constraints of DL Application in Clinical Settings

DL is still a long way from being able to function independently in the medical domain. Despite various successful DL model implementations, practical considerations should be recognised. Recent publications are experimental in nature and cannot be included in regular medical care practice, but they may demonstrate the potential and effectiveness of proposed detection/diagnostic models. In addition to this, it is difficult to reproduce the published works, because the codes and datasets used to train the models are generally not published. Additionally, in order to be efficient, the proposed methodology must be integrated into clinical information systems and Picture Archiving and Communications Systems. Unfortunately, till now, just a small amount of data has shown this type of interconnection. Additionally, demonstrating the safety of these systems to governmental authorities is a critical step toward clinical translation and wider use. Furthermore, there is no doubt that DL is rapidly progressing and improving.

In general, the new era of DL, particularly CNN, has effectively proved that they are more accurate and efficiently developed, with novel outcomes than the previous era. These methods have become diagnostically efficient, and in the near future, they are expected to surpass human experts. It could also provide patients with a more accurate diagnosis. Physicians must have a thorough understanding of the techniques employed in artificial intelligence in order to effectively interpret and apply it. Taking into consideration the obstacles in the way of clinical translation and various applications. These barriers range from proving safety to regulatory agency approval.

## 6. Future Aspect

For decades, people have debated whether or not to include DL technology in decision support systems. As the use of DL technology in radiology/orthopaedic traumatology grows, we expect there will be multiple areas of interest that will have significant value in the near future [48]. There is strong concurrence that incorporating DL into radiology/image-based professions would increase diagnostic performance [49,50]. Furthermore, there is consensus that these tools should be thoroughly examined and analysed before being integrated into medical decision systems. The DL-radiologist relationship will be a forthcoming challenge to address. Jha et al. [51] proposed that DL can be deployed for recurrent pattern-recognition issues, whereas the radiologists will focus on intellectually challenging tasks. In general, the radiologists need to have a primary knowledge and insight of DL and DL-based models/tools; although, the radiologists’ tasks would not be replaced by these tools and their responsibility would not be restricted to evaluating DL outcomes. These tools, on the other hand, can be utilised as a support system to verify radiologists’ doubts and conclusions. In order to integrate the DL–radiologists relationship, further studies need to be carried out on how we can train and motivate the radiologists to implement DL tools and evaluate their conclusions. DL technologies need to keep increasing their clinical application libraries. As illustrated in this article, several intriguing studies show how DL might increase the efficiency on clinical settings like fracture diagnosis on radiographs and CT scans, as well as fracture classifications and treatment decision assistance.

### 6.1. DL in Radiology Training

Radiologists’ expertise is the result of several years of practice in which the practitioner is trained to analyse a huge number of examinations by using a combination of literary and clinical knowledge. The dependency of interpretation skills is mainly on the quantitative as well as the accuracy of the radiographic diagnosis and prognosis. DL can analyse images using deep learning techniques and extract not only image cues but also numeric data, like imaging bio-markers or radiomic signatures that the human brain would not recognise. DL will become a component of our visual processing and analysis toolbox. During the training years if the software is introduced into the process of analysis, the beginners may not make enough unaided analysis, as a result, they may not develop adequate interpretation abilities. Conversely, DL will aid trainees in performing better diagnoses and interpretations. However, a substantial reliance on DL software for assistance by future radiologists is a concern with possible adverse repercussions. Therefore, the DL deployment in radiology necessitates that a beginner understands how to optimally incorporate DL in diagnostic practices, and hence a special DL and analytics course should be included in prospective radiology professional training curricula.

Another responsibility that must be accomplished is the initiative in training lawmakers and consumers about radiology, artificial intelligence, its integration, and the accompanying risks. In every rapid emerging industry, there is typically initial enthusiasm, followed by regret when early claims are not met. The hype about modern technology, which is typically driven by commercial interests, may claim more than what it can actually deliver and leads to play-down challenges. It is the duty of clinical radiologists to educate anyone regarding DL in radiology, and those who govern and finance our healthcare organisations, to establish a proper and secure balance saving patients while incorporating the greatest of new advancements.

### 6.2. Will DL Be Free to Hold the Position of a Radiologist?

It is a matter of concern among radiologists if DL be holding the position of a radiologist in future. However, this is not the truth. A very simple answer to this question is never. However, in the era of artificial intelligence, the professional lives of the radiologists will definitely change. With the use of DL algorithms, a number of regular tasks in the radiography process will be executed faster and more effectively. However, the radiologist’s job is a challenging one, as it involves resolving complicated medical issues [52]. Automatic annotation [53] can really help the radiologist in proper diagnosis. The real issue is not to resist the adoption and implementation of automation into working practices, but to accept the inevitable transformation in the radiological profession by incorporating DL into radiological procedures. One of the most probable risks is that “we will do what a machine urges us to do because we are astonished by the machines and rely on it to take crucial decisions.” This can be minimised if a radiologist train themselves and future associates about the benefits of DL, collaborate with researchers to ensure the proper, safe, meaningful, and useful deployment and assuring the usage is mainly for the benefit of the patients. As a matter of fact, DL may improve radiology and help clinicians to continuously increase their worth and importance.

Diagnosing a child X-ray, complex osteoporosis and bone tumour case remains as an intellectual challenge for the radiologist. It is difficult to analyse when the situation is complex and machines can assist a radiologist in proper diagnosis and can draw their attention to an ignored or complex case. For example, in the case of osteoporosis, machines can predict that this condition can lead to fracture in the future and precautions are to be taken. Another case is the paediatric x-rays, the bones of a child in the growing stage till the age of 15–18 years. This is very well understood by radiologists, but a machine can predict it as an abnormality. In this situation, radiologist expertise is required to take the decision whether the case is normal or not. Therefore, we can say that there is no point that DL will replace the radiologist, but they both can go hand in hand and support the overall decision system.

## 7. Conclusions

In this article, we have reviewed several approaches to fracture detection and classification. We can conclude that CNN-based models especially the InceptionNet and XceptionNet are performing very well in the case of fracture detection. Expert radiologists’ diagnosis and prognosis of radiographs is a labour-intensive and time-consuming process that could be computerised using fracture detection techniques. According to many of the researchers cited, the scarcity of the labelled training data is the most significant barrier to developing a high-performance classification algorithm. Still, there is no standard model available that can be used with the data available. We have tried to present the use of DL in medical imaging and how it can help the radiologist in diagnosing the disease precisely. It is still a challenge to completely deploy DL in radiology as there is a fear among them to lose their job. We are just trying to help the radiologist to assist them in their work and not to replace them.

## Figures and Tables

**Table 1:** Performance of various models for fracture detection.

No.	Author	Year	Modality	Model/Method	Skeletal Joints	Description	Performance
1	Kim et al. [30]	2018	Xray/MRI	Inception V3	Wrist	The author proved that the concept of transfer learning from CNNs in fracture detection on radiographs can provide the state of the performance.	AUC = 0.954 Sensitivity = 0.90 Specificity = 0.88
2	Olczak et al. [31]	2017	Xray/MRI	BVLC Reference CaffeNet network/VGG CNN/Network-in- network/VGG CNN S	Various Parts	Here, the research supports the use of deep learning to outperform the human performance.	Accuracy = 0.83
3	Cheng et al. [33]	2019	Radiographic images	DenseNet 121	Hips	The aim of this study was to localise and classify hip fractures using deep learning.	Accuracy ≈ 91 Sensitivity ≈ 98 Specificity ≈ 84
4	Chung et al. [32]	2018	Radiographic images	ResNet 152	Humeral	The authors proposed a model for the detection and classification of the fractures from AP shoulder radiographic images.	Accuracy ≈ 96 Sensitivity ≈ 0.99 Specificity ≈ 0.97 AUC ≈ 0.996
5	Urakawa et al. [10]	2018	Radiographic images	VGG_16	Hips	This study shows a comparison of diagnostic performance between CNNs and orthopaedic doctors.	Accuracy ≈ 95.5 Sensitivity ≈ 93.9 Specificity ≈ 97.40 AUC ≈ 0.984
6	Kitamura et al. [36]	2019	Radiographic images	7 modelsInception V3 ResNet (with/without drop&aux) Xception (with/without drop&aux) Ensemble A Ensemble B	Ankle	The study was done in order to determine the efficiency of CNNs on small datasets.	Best performance by Ensemble_A Accuracy ≈ 83 Sensitivity ≈ 80 Specificity ≈ 81
7	Yu [41]	2020	Radiographic images	Inception V3	hip	The proposed algorithm performed well in terms of APFF detection, but not so well in terms of fracture localization.	Accuracy = 96.9 AUC = 0.994 Sensitivity = 97.1 Specificity = 96.7
8	Gan [42]	2019	Radiographic images	Inception V4	Wrist	The authors implemented the algorithm for the detection of distal radius fractures.	Accuracy = 93 AUC = 0.961 Sensitivity = 90 Specificity = 96
9	Choi [43]	2019	Radiographic images	ResNet 50	Elbow	The authors aimed the development of dual input CNN-based deep learning model for automated detection of supracondylar fracture.	AUC = 0.985 Sensitivity = 93.9 Specificity = 92.2
10	Majkowska et al. [44]	2020	Radiographic images	Xception	Chest	The authors developed a model to detect opacity, pneumothorax, mass or nodule, and fracture.	AUC ≈ 0.86 Sensitivity = 59.9 Specificity = 99.4
11	Lindsey et al. [8]	2018	Radiographic images	Unet	wrist	This study involves the implementation of deep learning to help doctors to distinguish between fractured and normal wrist.	AUC = 97.5% Sensitivity = 93.9% Specificity = 94.5
12	Johari et al. [45]	2016	Radiographic images	probabilistic neural network (PNN) CBCT-G1/2/3, PA-G1/2/3	Vertical Roots	This study supports the initial detection of vertical roots fractures.	Best performance by PNN Model Accuracy ≈ 96.6 Sensitivity ≈ 93.3 Specificity ≈ 100
13	Heimer et al. [46]	2018	CT	deep neural networks.	Skull	The study aims at classification and detection of skull fractures curved maximum intensity projections (CMIP) using deep neural networks.	CMPIs THRESHOLD = 0.79 Specificity= 87.5 Sensitivity =91.4 CMPIs THRESHOLD = 0.75 Specificity= 72.5 Sensitivity =100
14	Wang et al. [11]	2022	CT	CNN	Mandibule	The author implemented a novel method for the classification and detection of mandibular fracture.	Accuracy = 90% AUC = 0.956
15	Rayan et al. [9]	2021	Radiographic images	XceptionNet	elbow	This study aims for a binomial classification of acute paediatric elbow radiographic abnormalities.	AUC = 0.95 Accuracy = 88% Sensitivity = 91% Specificity = 84%
16	Adam et al. [34]	2019	Radiographic images	AlexNet and GoogLeNet	femur	Here, the author aimed to evaluate the accuracy of DCNN for the detection of femur fractures.	Accuracy AlexNet = 89.4% GoogLeNet = 94.4%
17	Balaji et al. [35]	2019	x-ray	CNN based model	Diaphyseal Femur	In this study, the author implemented an automated detection and diagnosis of femur fracture.	Accuracy = 90.7% Specificity = 92.3% Sensitivity = 86.6%
18	Pranata et al. [37]	2020	Radiographic images	convolutional neural network (CNN)	Femoral neck	In this study, the author aimed at the detection of femoral neck fracture using genetic and deep learning methods.	Accuracy = 0.793 Specificity = 0.729 Sensitivity = 0.829
19	Rahmaniar et al. [26]	2019	CT	Computerised system	Calcaneal fractures	Here, the author aims at automated segmentation and detection of calcaneal fractures.	Accuracy = 0.86
20	Burns et al. [47]	2017	CT	Computerised system	spine	The author implemented a computerized system to detect classify and localize compression fractures.	
21	Tomita et al. [39]	2018	CT	Deep convolutional neural network	vertebra	This study aims at the early detection of osteoporotic vertebral fractures.	Accuracy = 89.2% F1 score = 90.8%
22	Muehlematter et al. [38]	2018	CT	Machine-learning algorithms	vertebra	Here, the author aims at evaluation of the performance of bone texture analysis with a machine learning algorithm.	AUC = 0.64

## Data Availability

Not applicable.
